# Intracorneal Ring Segment Implantation for the Management of Keratoconus in Children

**DOI:** 10.3390/vision5010001

**Published:** 2020-12-23

**Authors:** Pablo Larco, Pablo Larco, Daniel Torres, David P. Piñero

**Affiliations:** 1 Clínica Larco Visión, Quito 170135, Ecuador; plarco@icloud.com (P.L.); drpablolarco@gmail.com (P.L.J.); dantorres1983@gmail.com (D.T.); 2 Department of Optics, Pharmacology and Anatomy, University of Alicante, 03690 Alicante, Spain

**Keywords:** keratoconus, intracorneal ring segments, intrastromal ring segments, Keraring, corneal aberrations

## Abstract

The short-term safety and efficacy of intracorneal ring segment (ICRS) implantation in keratoconus eyes of children are investigated in this study. A retrospective interventional case series study including a total of 33 keratoconus eyes (age 8 to 17 years) that had undergone ICRS (Keraring segments, Mediphacos) implantation was conducted. Information about visual, refractive, pachymetric, corneal topographic and aberrometric, and corneal endothelial changes during a 3-month follow-up were extracted and analysed. A significant improvement was observed in logMAR corrected distance visual acuity (*p* = 0.005), combined with a statistically significant reduction in keratometric readings (*p* < 0.001). A reduction in the magnitude of corneal astigmatism of ≥1 D was observed in 52.8% of eyes. No significant changes were observed in corneal endothelial density (*p* = 0.317). Significant changes were found in the anterior vertical coma component (*p* = 0.002) as well as in the spherical aberration of the posterior corneal surface (*p* = 0.004). Only two relevant complications were described: one corneal microperforation with penetration of the ring segment into the anterior chamber (1 eye, 2.8%), and a case of ring extrusion (1 eye, 2.8%). ICRS implantation in children keratoconus eyes allows a reduction of corneal astigmatism, irregularity, and aberrations, leading to a significant visual improvement.

## 1. Introduction

Keratoconus is a degenerative corneal disease that is commonly manifested during puberty, with the possibility of progressing until the third or fourth decade of life [1]. This condition is characterized by a significant corneal thinning, corneal protrusion and induction of myopic astigmatism, with development of corneal opacity and hydrops in some cases [1]. In children, keratoconus is one of the most frequent causes leading to corneal transplantation with rates up to 20% [2,3]. It should be considered that keratoconus in children are commonly more severe, with fast progression and high incidence of pruritus associated [4,5,6]. Furthermore, children often manipulate their eyes with their fingers, and the medical accompaniment and care of family members in avoiding the factors that trigger the allergy is not always adequate. This mechanical stress on the cornea is a factor that may promote the progression and development of the disease [7].

Although keratoplasty is a potential solution in the most advanced keratoconus cases, both penetrating and deep lamellar keratoplasty are not exempt from complications such as rejection of the donor button, vascularization and infectious diseases [8]. In children, postoperative controls become more complex and with inadequate collaboration for suture removal, as additionally children are more prone to suffer traumas that can affect the integrity of the eye [8,9,10]. For this reason, the viability of other keratoconus treatment options in children are being investigated, such as corneal collagen crosslinking (CXL) [11]. Despite the proved efficacy and safety of intracorneal ring segments (ICRS) for the management of keratoconus [12], there are few studies evaluating the potential usefulness of this treatment option in children [13,14]. The aim of the current study was to evaluate the safety and efficacy of ICRS implantation in keratoconus in children, evaluating changes in visual acuity, refraction, pachymetry, corneal topography and aberrometry, and corneal endothelial density during a 3-month follow-up.

## 2. Materials and Methods

### 2.1. Patients

Retrospective interventional case series including a total of 33 keratoconus eyes (21 men and 12 women) that had undergone ICRS implantation using the femtosecond laser technology for the creation of the tunnels. All cases included were treated and followed at Clinica de Ojos Larco Vision (Quito, Ecuador), with all surgeries performed in the period from May 2015 to July 2018. Inclusion criteria were keratoconus diagnosis according to the standard criteria that consider the presence of the following signs: anterior corneal topographic asymmetric bowtie pattern, KISA ≥ 100, and one or more biomicroscopic keratoconus signs, such as Fleischer ring, significant corneal thinning, Vogt striae, conical protrusion on the cornea at the apex or anterior stromal scar [15]. Previous ocular surgery, corneal opacity affecting the visual axis and any other active ocular disease were considered as exclusion criteria. The study was conducted following the tenets of the Declaration of Helsinki and was approved by the ethics committee of the institution where the investigation was developed.

### 2.2. Clinical Examinations

A complete preoperative ophthalmological examination was performed in all cases including measurement of uncorrected (UDVA) and corrected distance visual acuity (CDVA), autorefraction (KR-800 Topcon Medical System), cycloplegic and manifest refraction, slit lamp evaluation of the anterior segment, fundus evaluation, pupillometry, specular microscopy for corneal endothelial cell density analysis (Konan Cell Check, Konan Medical USA Inc., Irvine, CA, USA) and corneal topographic, pachymetric and aberrometric evaluation with the Scheimpflug imaging-based system Pentacam (Oculus Optikgerate GmbH, Wetzlar, Germany). Postoperative examinations were performed the day after surgery, at 1 week, 1 month, and 3 months after surgery. In the last postoperative visit, a complete eye examination identical to the preoperative exam was performed. The importance of not rubbing the eyes and maintaining the anti-allergic treatment was remarked to all patients and their relatives, with the additional recommendation of attending to eye controls every 3 months.

All patients received indications and anti-allergic treatment prior to surgery and it was maintained indefinitely.

### 2.3. Surgery

All surgical procedures were performed by the same experienced surgeon (PL) under topical anaesthesia (combination of tetracaine 0.1% and oxybuprocaine 0.4%, Alcon, Fort Worth, TX, USA). The femtosecond laser platform FS 200 (Alcon Laboratories, Inc, Fort Worth, TX, USA) was used to create the corneal incision and tunnels. The tunnels were created at 70% of corneal depth ensuring that at least 100 μm of corneal tissue was present under the tunnel. Keraring segments (Mediphacos, Belo Horizonte, Brazil) were implanted in all cases, with a selection of the number of segments to implant as well as their diameter (SI5 or SI6), arc length and thickness according to the manufacturer’s nomogram and the scotopic pupil size measured. Table 1 summarizes the parameters used in the femtosecond laser platform for the different type of ring segments implanted. Topical antibiotics (Moxifloxacin 0.3%, Alcon Laboratories Inc., Fort Worth, TX, USA) were instilled at the time of implanting ICRS and at the end of the surgery. No stitches and/or therapeutic contact lenses were needed. A plastic protector was placed in the implanted eye to avoid the manipulation of the operated eye. Topical tobramycin 0.3% combined with sodium dexamethasone phosphate 0.1% (Tobradex, Alcon Laboratories Inc, Fort Worth, TX, USA) was instilled in the operated eye 4 times per day for 15 days.

### 2.4. Statistical Analysis

The statistical power associated to the sample size selected was calculated using the online calculator, https://www.imim.cat/ofertadeserveis/software-public/granmo/. Assuming a paired test in a same group for detecting significant differences in CDVA, the statistical power was 84.0% for the detection of a minimum difference of −0.16 logMAR (minimum change in CDVA observed at 3 months postoperatively), considering an alpha error of 0.05, the sample of 33 eyes, and the standard deviation of differences in CDVA at 3 months after surgery (0.31 logMAR).

Statistical analyses were performed with a commercially available software package (SPSS for Mac, v. 20.0; IBM Corporation, Armonk, NY, USA). Normality of data samples was evaluated by means of the Kolmogorov-Smirnov test. When parametric analysis was possible, the Student t test for paired data was used for comparisons between preoperative and postoperative visits, whereas the Wilcoxon ranked sum test was applied to assess the significance of such differences when parametric analysis was not possible. The Pearson or Spearman correlation coefficient was used to assess the level of correlation between preoperative corneal astigmatism and the clinical outcomes obtained depending if the condition of normality could be assumed or not. For all statistical tests, a *p*-value of less than 0.05 was considered as statistically significant.

## 3. Results

### 3.1. Description of the Sample

A total of 33 eyes of 33 patients with mean age of 14.9 years (SD: 1.9, median: 15.0, range: 8 to 17 years) were enrolled. The sample was comprised of 21 males (63.6%) and 12 females (36.4%). A total of 15 right (41.7%) and 21 left eyes (58.3%) were included. The model of ring segments SI5 was implanted in a total of 16 eyes (44.4%), whereas a total of 20 eyes (55.6%) were implanted with the SI6 ring segments. In all cases except in 12 eyes, two ring segments were implanted (a total of 60 ring segments), with the following distribution in terms of arc length: 90° in 11 eyes (11/60, 18.3%), 120° in 18 eyes (18/60, 30.0%), 150° in 15 eyes (15/60, 25.0%), 160° in 9 eyes (9/60, 15.0%), 210° in 4 eyes (4/60, 6.7%), and 340° in 3 eyes (3/60, 5%). Concerning ring segment thickness, the distribution of cases was as follows: 150 μm in 3 eyes (3/60, 5%), 200 μm in 14 eyes (14/60, 23.3%), 250 μm in 12 eyes (12/60, 20.0%), and 300 μm in 31 eyes (31/60, 51.7%).

### 3.2. Visual, Refractive, and Corneal Topographic Changes

**Figure 1:**
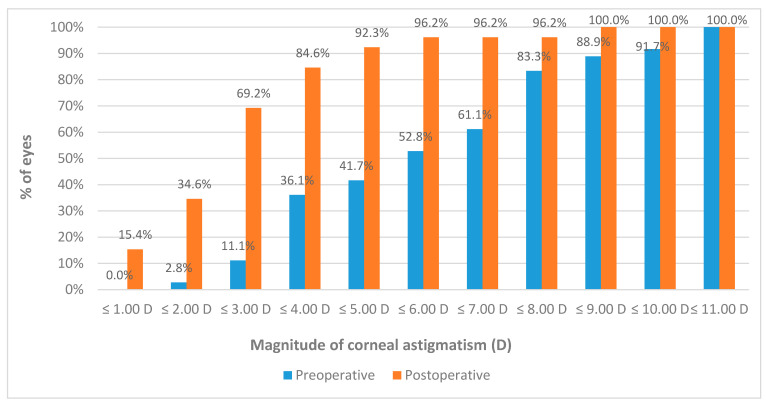
Preoperative and postoperative distribution of the magnitude of corneal astigmatism in the analyzed sample.

**Figure 2:**
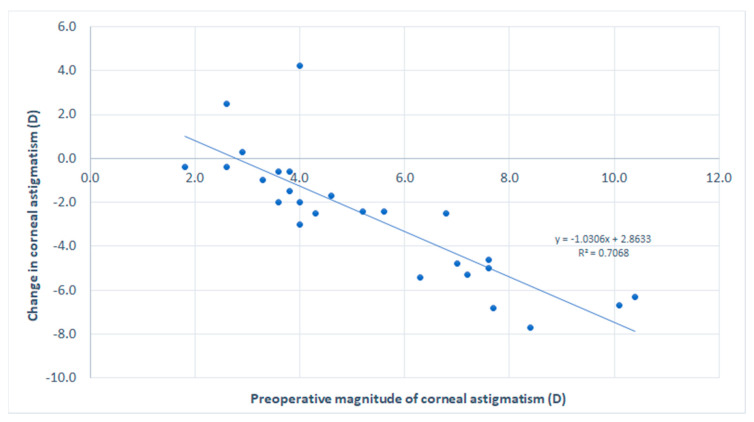
Scatterplot showing the relationship between the magnitude of preoperative anterior corneal astigmatism and the change induced at 3 months after surgery in anterior corneal astigmatism. The adjusting line to the data obtained by means of the least-squares fit is shown.

Table 2 summarizes the preoperative and postoperative visual, refractive and corneal tomographic data obtained in the analyzed sample. A reduction in magnitude was observed in sphere, cylinder, and spherical equivalent (SE) in most patients, but these changes did not reach statistical significance (*p* ≥ 0.066). However, a significant improvement was also observed in logMAR CDVA (*p* = 0.005). Concerning keratometric readings, a statistically significant reduction was observed in K1 (*p* = 0.001), K2 (*p* < 0.001), and KM (*p* < 0.001). Consequently, a significant reduction was observed in the magnitude of corneal astigmatism (*p* < 0.001), with 11.1% (4/36) and 69.2% (18/26) of eyes with magnitude of anterior corneal astigmatism of 3 D or less before and 3 months after surgery, respectively (Figure 1). A total of 52.8% (19/36) of eyes showed a reduction in the magnitude of anterior corneal astigmatism of 1 D or more. A very strong and significant correlation was found between the preoperative magnitude of anterior corneal astigmatism and the change induced at 3 months after surgery in anterior corneal astigmatism (r = −0.903, *p* < 0.001) (Figure 2). Concerning central pachymetry, no significant changes were observed with surgery (*p* = 0.647).

### 3.3. Corneal Aberrometric Changes

Table 3 summarizes the preoperative and postoperative corneal aberrometric data obtained in the analyzed sample. Statistically significant changes were found in the vertical coma component of the anterior corneal surface (*p* = 0.002) as well as the spherical aberration of the posterior corneal surface (*p* = 0.004). No significant changes were observed in the remaining aberrometric components (*p* ≥ 0.200). A poor although significant correlation was found between preoperative coma RMS and the change induced in this parameter with surgery (r = −0.443, *p* = 0.024). No correlation was found between the preoperative magnitude of spherical aberration and the change induced in this parameter with surgery (r = −0.185, *p* = 0.336).

### 3.4. Complications

**Figure 3:**
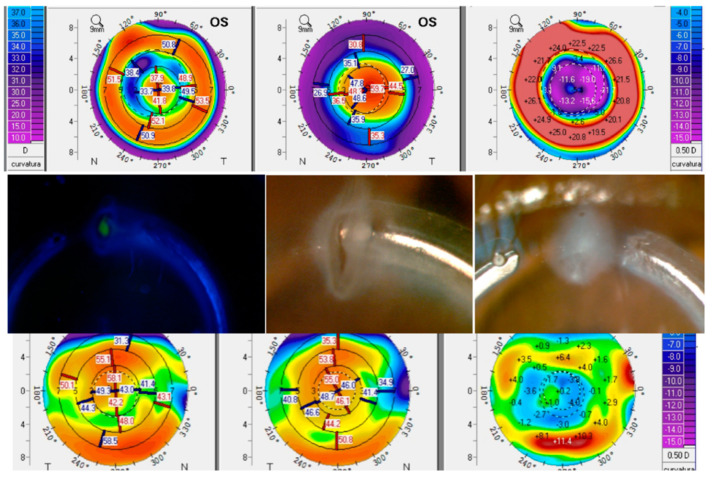
Graphic summary of changes occurring in the eye suffering a ring extrusion. **Up**: topographic modifications achieved with the ring segment (left: post, center: pre, right: change), showing below the corresponding slit lamp images. **Down**: topographic changes during 30 days of follow-up after explanting the ring segment (left 30 days post-explantation, middle: the same day after explantation, right: change).

Concerning safety and complications, only two relevant complications were described, one corneal microperforation with penetration of the ring segment into the anterior chamber (1 eye, 2.8%), and a case of ring extrusion (1 eye, 2.8%) (Figure 3). No significant changes were found in corneal endothelial density (*p* = 0.317), with mean preoperative and 3-month postoperative values of 2795.08 cell/mm^2^ (SD: 304.53, median: 2857, range: 1869 to 3205) and 2624.50 cell/mm^2^ (SD: 512.42, median: 2822, range: 1869 to 2822), respectively.

## 4. Discussion

In the last two decades, advances in keratoconus management have been focused on avoiding corneal transplantation [16]. Although the prognosis of keratoplasty has been shown to be good in keratoconus, this procedure is more complex in children as the cornea is thinner and less rigid compared to adults, with more significant inflammatory reaction and more risks of infection and corneal graft rejection [10]. Several studies have confirmed the validity of CXL [11] and contact lenses fitting of special designs, such as scleral lenses [17], for the management of keratoconus, even in children [18,19,20,21]. Likewise, some studies have shown the potential of the use of ICRS in keratoconus in children, allowing a regularization of the corneal surface, an improvement of visual quality and even an improvement in contact lens tolerance [13,14,22,23]. In most of the cases, this implantation was combined with CXL [22,23]. The aim of the current study was to analyze the short-term visual, refractive, and corneal aberrometric outcomes after ICRS implantation for the management of keratoconus in a sample of children. Thus, the real effect of these implants in this type of corneas can be clearly characterized and understood independently if CXL is applied afterwards to obtain a better control of the progression of the disease.

Concerning the refractive outcomes, a reduction in magnitude was observed in sphere, cylinder and SE in most of patients, but not reaching statistical significance in the overall sample. This is consistent with the variability of ICRS outcomes reported in the peer-reviewed literature according to the corneal topographic phenotypic profile and the level of severity of the disease [24,25,26]. Recently, Sedaghat and colleagues [27] confirmed in adult patients implanted with the same type of ICRS (KeraRing) that a greater difference between the preoperative uncorrected and corrected distance visual acuity and more coincidence of the most elevated points in the two corneal surfaces on the elevation maps increased the rate of successful outcome. Despite the non-statistically significant change in refraction, significant modifications were found in the current series in keratometric readings, the magnitude of corneal astigmatism, and logMAR CDVA, as in previous series using the same and other type of ICRS in adults and children [12,13,14]. Specifically, significant central corneal flattening, reduction of magnitude of astigmatism and improvement of CDVA were observed, with 52.8% of eyes showing a reduction in the magnitude of anterior corneal astigmatism of 1 D or more. Heikal et al. [28] found in a sample of 20 adult keratoconus eyes implanted with KeraRing using the femtosecond laser technology that mean LogMAR CDVA improved from 0.85 ± 0.17 preoperatively to 0.26 ± 0.11 at 3 months postoperatively (*p* = 0.001). Alfonso et al. [13] reported in a sample of 118 eyes of 88 children a change of mean CDVA from 0.67 ± 0.37 logMAR preoperatively to a value of 0.37 ± 0.30 logMAR at 6 months after ICRS implantation. In the current series, a change from a mean preoperative value of 0.86 ± 0.67 logMAR to a mean 3-month postoperative value of 0.30 ± 0.32 logMAR was observed. Gharaibeh et al. [29] also reported a significant visual improvement after KeraRing implantation in a sample of 55 eyes of adult patients, with a decrease in mean keratometry from 51.83 ± 4.14 D preoperatively to 47.27 ± 3.68 D at 6 months after surgery that is consistent with the keratometric change found in the current study.

The change induced at 3 months postoperatively in the magnitude of anterior corneal astigmatism was strongly correlated with the preoperative magnitude of anterior corneal astigmatism. This correlation was negative which indicated that less reduction of corneal astigmatism was achieved in those cases with lower levels of baseline corneal toricity. This suggests that no more undercorrection was present in cases of high levels of corneal astigmatism. However, despite the greater astigmatic corrective effect achieved in very toric corneas, the impact of potential misalignments of the meridian of correction is higher, as demonstrated in previous series [30]. In any case, improvements in nomograms in the last years have allowed the clinician to obtain more predictable outcomes by selecting a more optimized position of ICRS, even in cases of high astigmatism [25,26].

The analysis of changes in corneal aberrations in the current series revealed that statistically significant changes were found in the vertical coma component of the anterior corneal surface as well as in the spherical aberration of the posterior corneal surface. There was a trend to a reduction of primary coma RMS that did not reach statistical significance, as has been reported in other studies evaluating the corneal aberrometric outcomes after KeraRing implantation [31]. The main factor accounting for this finding may be the great variability of corneal high order aberrations that was present in the sample evaluated as cases with different levels of severity were included. A similar situation was reported by Haddad et al. [32] when comparing the outcomes of two different types of ICRS, being one of them KeraRing. It should be considered that corneal high order aberrations increase with the level of severity [13], and the efficacy of ring segments is conditioned by this aberrometric profile [31]. Possibly, significant changes in primary coma RMS would have been detected if a larger sample would have been evaluated and subdivided by severity groups. Indeed, Alfonso et al. [13] reported a statistically significant decrease in coma-like RMS in a sample including 118 eyes from 88 pediatric patients undergoing ICRS implantation. Besides primary coma, a significant change of primary spherical aberration towards a more positive value was found as in previous series [31,32]. This finding is consistent with the curvature change induced in the central part of the cornea (central flattening vs. peripheral steepening), leading to a less prolate corneal configuration.

Finally, the safety of the procedure was confirmed with the gain of lines of CDVA and the presence of only two relevant complications, a corneal microperforation with penetration of the ring segment into the anterior chamber and a ring extrusion. These complications have been also reported in series with adult patients [12]. In our series, the microperforation occurred in a 13-year-old boy at 3 days after surgery. Due to eye rubbing, the ring segment penetrated the anterior chamber and it was then explanted, with the implantation of ring segment of less arc length afterwards to avoid the contact with the compromised area. The ring extrusion occurred in an 8-year-old girl implanted with a 340° ring segment and with association to a significant foreign body sensation (Figure 3). The ring segment was fractioned manually by cutting one end and was re-inserted away from the incision, disappearing the discomfort. This case presented a favourable evolution and no topographic changes were observed in its follow-up of 31 months. In previous series showing the results of ICRS in children, only one complication was reported in one case: ring segment explantation 2 years after its implantation due to vascularization and corneal thinning [23]. Concerning pachymetry and corneal endothelial density, no significant changes were detected during the follow-up, confirming the safety of the procedure.

As limitations of the study, it should be noted that no validated questionnaires were used to assess and characterize changes in symptomatology. This should be investigated in future series evaluating the impact of ICRS implantation in children with keratoconus. Likewise, the results of this technique in a longer follow-up period should be reported to confirm the long-term safety of the procedure. Despite these limitations, this article adds new information in terms of tomographic and corneal aberrometric changes to the currently available peer-reviewed literature [13,14,33] about the clinical outcomes of ICRS information in pediatric keratoconus.

## 5. Conclusions

In conclusion, the implantation of ICRS in children is a safe procedure, which is not exempt from complications like the two reported cases, related to manipulation of the eyes, despite having been warned not to do it and that they were solved without problems. The case of the migration of the ICRS to the anterior chamber had a previous history of micro perforation 3 months before the implantation of the ICRS. Possibly, the combination with CXL is necessary in most cases to avoid keratoconus progression, but more studies are still needed to confirm this and to evaluate the safety and efficacy of this combination. Abreu et al. [14] reported signs of keratoconus progression in children implanted with ICRS and followed more than 6 years. In keratoconus cases in children in whom their clinical picture is more aggressive, the treatment must comprise three phases which are: preventing progression, regularizing the cornea, and rehabilitating vision. ICRS can contribute to improving vision and regularize the cornea and defer or avoid a corneal transplant. It should be considered that although corneal collagen cross-linking stops the progression of keratoconus, visual impairment persists and ICRS can be a valid alternative with the optimization of the femtosecond laser procedure to regularize the cornea, improve vision, facilitate the fitting of contact lenses, and to avoid or defer corneal transplantation.

## Figures and Tables

**Table 1:** Parameters used in the femtosecond laser platform for the different type of ring segments implanted.

Parameters	SI5 (90°–120°–160°)	SI5 (210°)	SI6 (90°–120°–160°)	SI6 (210°)	355°
Inner diameter	5.0 mm	5.0 mm	5.8 mm	5.8 mm	5.2 mm
Outer diameter	5.9 mm	6.0 mm	7.0 mm	7.1 mm	6.6 mm
Incision	1.4 × 1.4 mm	1.4 × 1.4 mm	1.5 × 1.5 mm	1.5 × 1.5 mm	1.5 × 1.5 mm
Depth	75% of the thinnest pachymetry in a 5-mm zone	75% of the thinnest pachymetry in a 5-mm zone	75% of the thinnest pachymetry in a 6-mm zone	75% of the thinnest pachymetry in a 6-mm zone	75% of the thinnest pachymetry in a 5-mm zone

**Table 2:** Summary of preoperative and postoperative visual, refractive and corneal tomographic data in the analyzed sample. In each box, the mean (standard deviation) is provided and below the median (range). The corresponding *p*-values for the comparison between preoperative and postoperative visits are shown for each parameter evaluated.

Mean (SD)	Preoperative	Postoperative	*p*-Value
Median (Range)			
Sphere (D)	−3.17 (4.82)	−2.75 (5.76)	0.655
	−1.38 (−20.50 to 0.75)	0.00 (−15.75 to 3.00)	
Cylinder (D)	−5.02 (2.34)	−1.70 (2.11)	0.066
	−4.63 (−9.75 to −1.50)	−0.50 (−5.00 to 0.00)	
Spherical equivalent (D)	−5.68 (5.17)	−3.60 (6.45)	0.066
	−4.00 (−25.38 to −0.25)	0.00 (−18.25 to 0.62)	
LogMAR CDVA	0.86 (0.67)	0.30 (0.32)	0.005
	0.70 (0.00 to 3.00)	0.29 (0.00 to 1.00)	
K1 (D)	49.53 (5.48)	48.28 (5.35)	0.001
	47.90 (42.20 to 63.70)	47.45 (40.20 to 59.40)	
K2 (D)	55.38 (5.56)	50.98 (5.71)	<0.001
	54.25 (46.90 to 67.70)	49.50 (43.50 to 65.90)	
KM (D)	52.27 (5.39)	49.58 (5.46)	<0.001
	50.90 (45.60 to 65.70)	48.35 (42.30 to 61.50)	
Anterior corneal astigmatism (D)	5.85 (2.38)	2.70 (1.56)	<0.001
	5.65 (1.80 to 10.40)	2.45 (0.70 to 8.20)	
CCT (μm)	451.42 (49.86)	445.77 (59.71)	0.647
	460.50 (335 to 539)	465.00 (285 to 536)	

**Table 3:** Summary of preoperative and postoperative corneal aberrometric data in the analyzed sample. The corresponding *p*-values for the comparison between preoperative and postoperative visits are shown for each parameter evaluated.

Mean (SD)	Preoperative	Postoperative	*p*-Value
Median (Range)			
**Anterior corneal surface:**			
Z_3_^−1^ (μm)	−0.89 (1.16)	−1.00 (1.48)	0.002
	−0.78 (−4.28 to 1.70)	−1.04 (−3.98 to 2.74)	
Z_3_^1^ (μm)	−0.08 (1.74)	0.02 (1.23)	0.395
	0.16 (−5.84 to 3.04)	0.17 (−1.90 to 3.14)	
Coma RMS (μm)	1.94 (1.15)	1.88 (1.02)	0.485
	1.77 (0.17 to 6.21)	1.62 (0.49 to 3.98)	
Z_4_^0^ (μm)	−0.99 (0.94)	−0.97 (1.07)	0.713
	−0.89 (−3.82 to 1.18)	−0.90 (−4.04 to 0.60)	
**Posterior corneal surface:**			
Z_3_^−1^ (μm)	0.24 (0.33)	0.21 (0.34)	0.617
	0.25 (−0.37 to 1.19)	0.18 (−0.43 to 1.09)	
Z_3_^1^ (μm)	0.01 (0.41)	−0.01 (0.48)	0.909
	−0.08 (−0.70 to 1.32)	−0.01 (−1.04 to 0.90)	
Coma RMS (μm)	0.50 (0.29)	0.54 (0.30)	0.501
	0.43 (0.12 to 1.56)	0.49 (0.04 to 1.24)	
Z_4_^0^ (μm)	0.14 (0.18)	0.24 (0.19)	0.004
	0.11 (−0.15 to 0.65)	0.21 (−0.21 to 0.61)	
**Total cornea:**			
Z_3_^−1^ (μm)	−0.75 (1.05)	−0.94 (1.53)	0.431
	−0.69 (−3.80 to 1.66)	−0.97 (−4.78 to 2.78)	
Z_3_^1^ (μm)	−0.04 (1.59)	0.02 (1.00)	0.454
	0.32 (−5.49 to 2.89)	0.10 (−1.68 to 2.72)	
Coma RMS (μm)	1.72 (1.08)	1.73 (1.06)	0.381
	1.48 (0.22 to 5.76)	1.35 (0.36 to 4.79)	
Z_4_^0^ (μm)	−0.96 (1.61)	−0.71 (1.03)	0.200
	−0.52 (−8.82 to 1.54)	−0.72 (−3.75 to 0.79)	

## Data Availability

Data available on request due to privacy restrictions.

## Abbreviations

The following abbreviations are used in this manuscript:

SD	standard deviation
D	diopters
UDVA	uncorrected distance visual acuity
CDVA	corrected distance visual acuity
K1	flattest keratometric reading
KM	mean keratometric reading
CCT	central corneal thickness
